# Loss of PI3k activity of inositol polyphosphate multikinase impairs PDK1-mediated AKT activation, cell migration, and intestinal homeostasis

**DOI:** 10.1016/j.isci.2023.106623

**Published:** 2023-04-11

**Authors:** Luke Reilly, Evan R. Semenza, George Koshkaryan, Subrata Mishra, Sujan Chatterjee, Efrat Abramson, Pamela Mishra, Yoshitasu Sei, Stephen A. Wank, Mark Donowitz, Solomon H. Snyder, Prasun Guha

**Affiliations:** 1 The Solomon H. Snyder Department of Neuroscience, Johns Hopkins University School of Medicine, Baltimore, MD 21205, USA; 2 Department of Neurology, Johns Hopkins University School of Medicine, Baltimore, MD 21205, USA; 3 Digestive Diseases Branch, National Institute of Diabetes and Digestive and Kidney Diseases, National Institutes of Health, Bethesda, MD, USA; 4 Department of Medicine, Division of Gastroenterology, Johns Hopkins University School of Medicine, 720 Rutland Avenue, Baltimore, MD 21205, USA; 5 Department of Psychiatry and Behavioral Sciences, Johns Hopkins University School of Medicine, Baltimore, MD 21205, USA; 6 Department of Pharmacology and Molecular Sciences, Johns Hopkins University School of Medicine, Baltimore, MD 21205, USA; 7 Department of Biophysics and Biophysical Chemistry, Johns Hopkins University School of Medicine, Baltimore, MD 21205, USA; 8 Reference Standard Laboratory, United States Pharmacopeial Convention, Rockville, MD 20852, USA; 9 Nevada Institute of Personalized Medicine (NIPM), University of Nevada, Las Vegas, NV 89154, USA; 10 School of Life Sciences, University of Nevada, Las Vegas, NV 89154, USA; 11 Marie-Josée and Henry R. Kravis Center for Molecular Oncology, Memorial Sloan Kettering Cancer Center, New York City, NY, USA

**Keywords:** Gastroenterology, Molecular biology, Cell biology

## Abstract

Protein kinase B (AKT) is essential for cell survival, proliferation, and migration and has been associated with several diseases. Here, we demonstrate that inositol polyphosphate multikinase (IPMK’s) lipid kinase property drives AKT activation via increasing membrane localization and activation of PDK1 (3-Phosphoinositide-dependent kinase 1), largely independent of class I PI3k (cPI3K). Deletion of IPMK impairs cell migration, which is partially associated with the abolition of PDK1-mediated ROCK1 disinhibition and subsequent myosin light chain (MLC) phosphorylation. IPMK is highly expressed in intestinal epithelial cells (IEC). Deleting IPMK in IEC reduced AKT phosphorylation and diminished the number of Paneth cells. Ablation of IPMK impaired IEC regeneration both basally and after chemotherapy-induced damage, suggesting a broad role for IPMK in activating AKT and intestinal tissue regeneration. In conclusion, the PI3k activity of IPMK is necessary for PDK1-mediated AKT activation and intestinal homeostasis.

## Highlights

• PI3k activity of IPMK is critical for PDK1 membrane localization
• IPMK is important for AKT activation
• IPMK is crucial for cell migration
• IPMK is linked to intestinal regeneration/Paneth cell organization

## Graphical abstract

**Figure:**
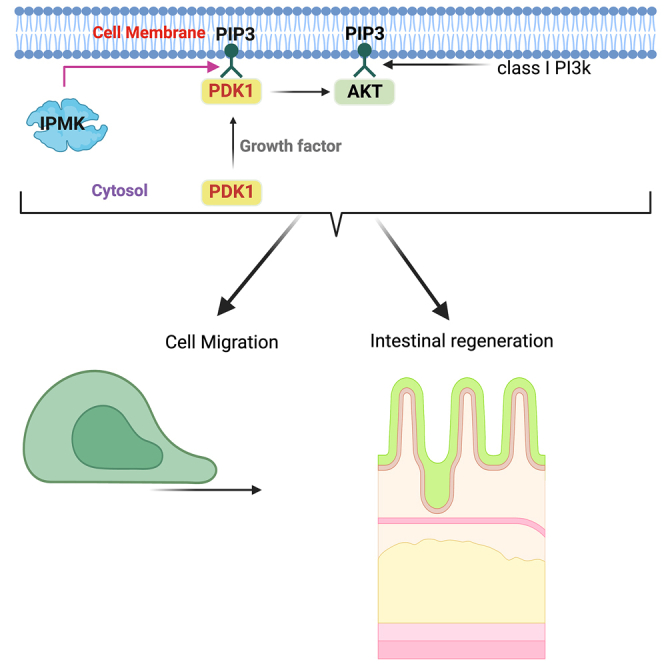

## Introduction

Inositol polyphosphates are signaling molecules generated by an evolutionarily conserved family of kinases. Inositol 1,4,5-trisphosphate (IP_3) is well established as a physiologic regulator of intracellular calcium.^1^ Among the family of kinase enzymes regulating inositol phosphates, IPMK is notable for its generation of multiple inositol phosphates, including inositol tetrakisphosphate (IP_4) and inositol pentakisphosphate (IP_5).^2^ Additionally, IPMK serves as a phosphoinositide 3-kinase (PI3k) in mammalian cells.^3^ The PI3k activity of IPMK, like that of cPI3k enzymes, contributes to AKT activation.^3^ Independent of its kinase activity, IPMK mediates autophagy by binding to AMP-dependent protein kinase and the autophagy-activating kinase ULK1.^4^ IPMK also participates in nutrient sensing and activation of mTORC1 and mTORC2.^5^ Epidemiological studies indicate that the mutation of IPMK at its nuclear localization signal is associated with familial intestinal carcinoids.^6^ Genome-wide association study (GWAS) data from patients with inflammatory bowel disease (IBD) have established IPMK as a putative risk gene.^7^

AKT is a central player in the regulation of metabolism, cell survival, motility, transcription, and cell-cycle progression.^8^ cPI3k (p110 α,β,γ,andδ) generates the second messenger phosphatidylinositol (3,4,5)-trisphosphate (PIP_3) from phosphatidylinositol (4,5)-bisphosphate (PIP_2). PIP_3 production stimulates the translocation of AKT from the cytoplasm to the plasma membrane, an action that requires its pleckstrin homology (PH) domain. The serine/threonine kinase phosphoinositide-dependent kinase 1 (PDK1) also contains a PH domain and is recruited to the plasma membrane by PIP_3. PDK1 phosphorylates AKT at Thr308,^8^ while phosphorylation at Ser473 is mediated by mTORC2.^9^ Phosphorylation of AKT at both Thr308 and Ser473 generates an active form of AKT, which targets specific proteins in the cytoplasm and nucleus. Several lines of evidence suggest that cPI3k is not the critical enzyme for recruiting PDK1 and mTORC2 to the membrane to activate AKT.^10^^,^^11^^,^^12^ PIP_3-mediated PDK1 membrane localization is also crucial for the activation of Rho-associated coiled-coil-containing kinase 1 (ROCK1), an essential mediator of cell migration. PDK1-mediated ROCK1 activation appears to be only marginally influenced by the inhibition of cPI3k,^12^^,^^13^ suggesting a different PI3k mediates this effect. We previously showed that the deletion of IPMK abolishes the activation of AKT^3^ via heretofore obscure mechanisms. Here we demonstrate that IPMK is the principal PI3k regulating PDK1’s membrane localization and associated AKT activation and ROCK1-mediated cell migration. We further show that IPMK physiologically influences the self-renewal of the intestinal epithelium.

## Results

### Inositol polyphosphate multikinase -mediated AKT activation is independent of cPI3k

**Figure 1:**
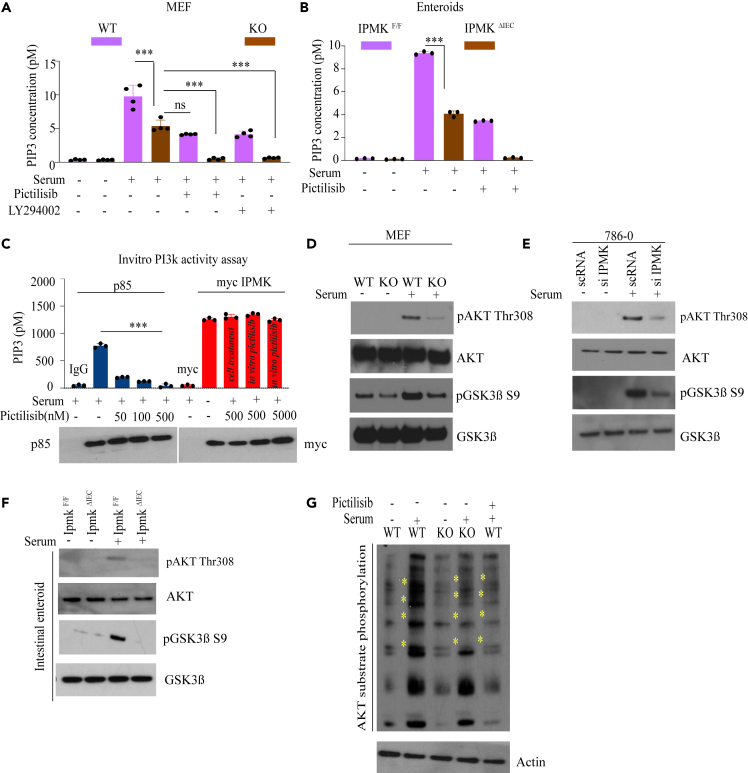
IPMK mediates AKT activation (A) Total PIP_3 concentration in MEF cells by PIP_3 ELISA. ∗∗∗p < 0.001. Data are graphed as mean ± SD. (B) PIP_3 concentration in intestinal enteroids by PIP_3 ELISA. ∗∗∗p < 0.001. Data are graphed as mean ± SD. (C) P85 and myc IPMK were immunoprecipitated and used *in vitro* PI3k assays by ELISA. Immunoblot of p85 and myc to confirm the total amount of protein immunoprecipitated. Pictilisib treatment in different doses (nM). ∗∗∗p < 0.001. Data are graphed as mean ± SD. (D) Western blot of AKT (Thr308) and GSK3β (Ser9) phosphorylation in serum-starved WT and IPMK KO MEFs with or without serum stimulation for 5 min. ( n=3). (E) Western blot of AKT (Thr308) and GSK3β (Ser9) phosphorylation in 786-0 cells after knocking down IPMK by siRNA. ( n=3). (F) Western blot of AKT (Thr308) and GSK3β (Ser9) phosphorylation in intestinal enteroids. (n= 3). (G) Western blot of phosphorylated AKT substrates in WT and IPMK KO MEFs treated with serum or pictilisib (100 nM, 30 min). (n= 3).

Previously, we showed that IPMK is a novel eukaryotic PI3kinase, and deletion of IPMK impairs the activation of AKT.^3^ Here we investigated the physiological importance of IPMK as a lipid kinase and its detailed mechanism of AKT activation. To determine the contribution of IPMK to the total cellular PIP_3 pool, we used a PIP_3 Mass ELISA kit. Using IPMK WT/KO MEFs, we found that overnight serum starvation markedly diminished intracellular PI (3,4,5) P3 levels in both WT/KO cells (Figures 1 and S1F). PIP_3 is rapidly produced with in 5 min of serum treatment. Deletion of IPMK diminished the PIP_3 level by around 44.9%. The inhibition of cPI3k with two selective inhibitors, Pictilisib (GDC0941, a pan cPI3k inhibitor with the highest selectivity at the concentration of 3-33 nM *in vitro* and used in clinical trial phase Ib) and LY294002, reduced PIP_3 level by 58% in WT cells. Interestingly, even after cPI3k inhibitor (cPI3ki) treatment, WT cells retain almost 42% of PIP_3 , implying the presence of a cPI3k independent PIP_3 pool (Figure 1A). IPMK KO cells treated with cPI3ki had further reduced PIP_3 levels which were comparable to serum-starved cells (Figure 1A). This suggests lipid kinases generating PIP_3 in IPMK KO cells are sensitive to cPI3ki. Intracellular PIP_2 levels were comparable in WT/KO cells (Figure S1A), demonstrating that the production of PIP_3 in IPMK KO cells was not influenced by the PIP_2 pool.

To examine whether loss of IPMK diminished PIP_3 production in primary cells, we generated the intestinal deletion of IPMK (*Ipmk*^*ΔIEC*^) as IPMK is highly enriched in intestinal crypts (Figures 1B and S1F). We purified primary crypts to perform the enteroid culture. Consistent with MEF data, PIP_3 production was substantially reduced in *Ipmk*^*ΔIEC*^ crypt compared to *Ipmk* ^*F/F*^, suggesting that PI3kinase activity of IPMK is essential for PIP_3 production in primary intestinal enteroid cells (Figure 1B). Intracellular PIP_2 levels were comparable in *Ipmk* ^*F/F*^/*Ipmk*^*ΔIEC*^ cells (Figure S1B).

To delineate the biological importance of the lipid kinase activity of IPMK, it is critical to understand the influence of cPI3k or PI3ki on IPMK’s lipid kinase activity. Previously, Resnick et al.^14^; showed that recombinant rat and yeast IPMK protein purified from untreated cells was insensitive to wortmannin treatment. However, Maag et al.^3^ showed that IPMK purified from wortmannin-treated cells partially lost its PI3kinase activity, but was ineffective when treated *in vitro*, directly in an IPMK’s lipid kinase reaction. Later it was discovered that wortmannin is not a specific cPI3ki, and it directly inhibits multiple essential kinases such as ATM, ATR, MLCK, and so forth,^15^ which may have off-target severe effects when treated in cell lines.

To determine the influence of cPI3ki on IPMK, we overexpressed myc-IPMK in HeLa cells, followed by immunoprecipitation using myc agarose beads to perform *in vitro* PI3kinase activity assay of IPMK (Figure 1C). Interestingly, IPMK purified from pictilisib-treated cells completely retained its PI3kinase activity, insinuating that cPI3k does not influence IPMK’s lipid kinase property (Figure 1C). Furthermore, we purified IPMK and p85 from untreated cells and incubated immunocomplex with Pictilisib *in vitro*. As expected, treatment with Pictilisib markedly diminished PI3kinase activity of the p85 immunocomplex, whereas there was no effect on IPMK’s PI3kinase activity even at the high dose of 5 μM (Figure 1C). IPMK purified from serum-starved cells also showed substantial PI3kinase activity, implying that serum or growth factor stimulation mediated posttranslational modification on IPMK is important for its lipid kinase activity in the cellular system but not essential for its *in vitro* kinase activity (Figure 1C). Previous studies demonstrated that other PI3kinase inhibitors, such as LY294002 and Wortmannin, do not influence IPMK’s lipid kinase activity *in vitro*,^14^ which agrees with our present finding and confirms that the lipid kinase activity of IPMK is independent of cPI3ki.

To ascertain the influence of IPMK on the lipid kinase activity of cPI3k, we immunoprecipitated the p85/p110 complex from IPMK WT and IPMK KO MEFs, followed by analyzing the lipid kinase activity of cPI3k using a PI3kinase activity ELISA. Intriguingly, p85/p110 immunocomplex purified from IPMK KO MEFs and WT cells displayed comparable activity, confirming that cPI3k activity is independent of IPMK (Figure S1C).

Overall, the above data conclude that IPMK is a physiological PI3kinase, and its lipid kinase activity is independent of cPI3k and entirely unresponsive to cPI3ki.

Activation of AKT is highly regulated by PIP_3 production.^16^ PDK1 and mTORC2 are two major kinases phosphorylating AKT at Thr308 and Ser473, respectively.^16^ We previously showed that IPMK directly promotes mTORC2 activation^5^ and thus may impair AKT Ser473 phosphorylation (Figures S1D and S1E). Here, we explored mechanisms underlying deficits in AKT Thr308 phosphorylation in *Ipmk*-deleted cells. In agreement with the previous study,^3^ we found that *Ipmk* deletion markedly diminished AKT phosphorylation at Thr308 in MEF (Figures 1D and S1F). We extended our findings to other cell lines. Depletion of IPMK in 786-0 (a renal cancer cell lines) substantially reduced AKT Thr308 phosphorylation (Figures 1E and S1F). Next, we analyzed the effects of IPMK loss in intestinal enteroid culture. Deletion of IPMK markedly diminished AKT Thr308 phosphorylation in *Ipmk*^*ΔIEC*^ enteroids compared to *Ipmk*^*F/F*^ (Figures 1F and S1F), suggesting a cell line independent influence of IPMK on AKT Thr308 phosphorylation. AKT phosphorylates GSK3β at Ser9 to inactivate GSK3β.^8^ IPMK KO MEFs showed a substantial loss of GSK3β phosphorylation at Ser9 in all experimental cell types (Figures 1D–1F). To determine whether the phosphorylation of other AKT substrates might be defective in IPMK-deleted cells, we used an antibody that recognizes proteins containing phosphorylated AKT substrate domains.^17^ Phosphorylation detected by this antibody was considerably reduced in IPMK KO MEFs and comparable to cells treated with the pan-cPI3ki pictilisib (Figure 1G).

Hence, we conclude that the lipid kinase activity of IPMK is independent of cPI3k and is critical for AKT activation.

### PI3k activity of inositol polyphosphate multikinase is required for the activation of AKT but not AKT membrane localization

**Figure 2:**
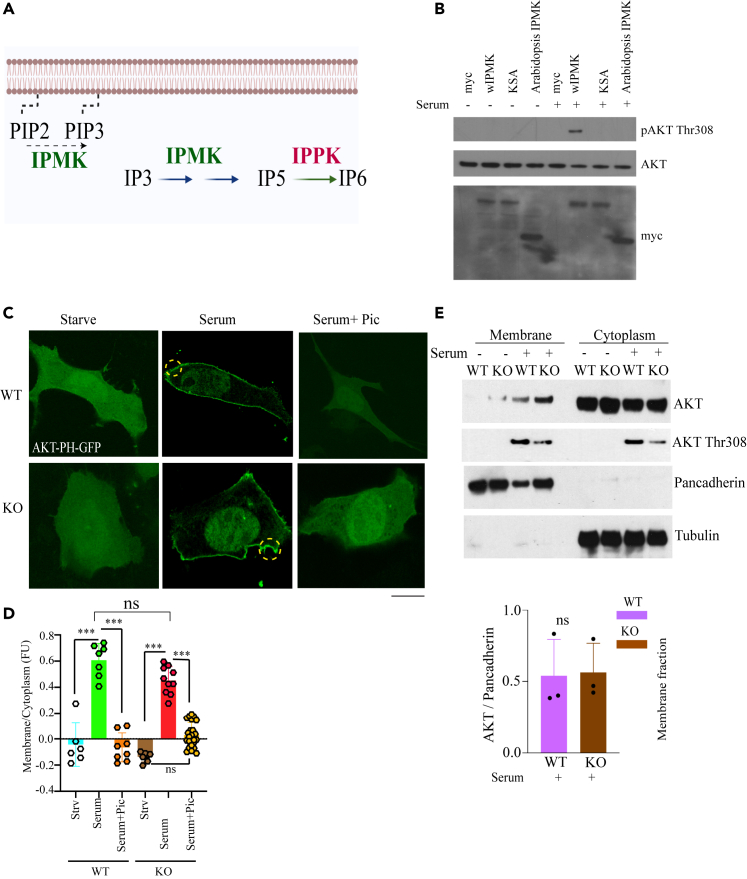
PI3k activity of IPMK is required for the activation of AKT (A) Schematic diagram of IPMK enzymatic pathway. (B) Western blot of AKT Thr308 phosphorylation in response to serum stimulation in IPMK KO MEFs stably overexpressing myc-tagged empty vector (myc), WT IPMK (WT), or kinase dead IPMK (KSA) and Arabidopsis IPMK. (C) AKT-PH-GFP transfected in IPMK WT/KO MEF and performed confocal imaging for AKT-PH-GFP membrane localization. Scale bar 20 μm. Membrane-localized AKT-PH-GFP is marked by a yellow circle. (D) The bar diagram shows the relative AKT-PH-GFP membrane/cytoplasm fluorescence ratio. ∗∗∗p < 0.001. Data are graphed as mean ± SD. (E) The cell fractionation study to understand endogenous AKT membrane localization in MEFs. Pancadherin is the marker for cell membrane fraction, and tubulin for the cytosolic fraction. ns = not significant.

To ascertain the importance of IPMK’s catalytic activity (Figure 2A) in regulating AKT phosphorylation, we rescued IPMK KO MEFs with stable overexpression of WT or kinase-dead (KSA) IPMK and *Arabidopsis* IPMK (IPK2β).^3^ We previously showed that IPK2β possesses only inositol kinase property but lacks lipid kinase activity.^3^ IPK2β fails in rescuing AKT Thr308 activation, confirming that the PI3kinase activity of IPMK is essential for AKT Thr308 phosphorylation (Figure 2B).

PIP_3 promotes AKT membrane localization and subsequent activation.^8^ To investigate the role of IPMK in growth factor-stimulated membrane localization of AKT, we overexpressed the AKT PH domain fused to GFP in WT and IPMK KO MEFs. Serum stimulation efficiently promoted GFP-AKT-PH membrane translocation in WT and KO cells (Figures 2C and 2D). By contrast, GFP-AKT-PH membrane localization was prevented by treatment with pictilisib (pan-cPI3ki). These immunocytochemical results were further confirmed by cell fractionation and western blotting of AKT in the cytoplasmic and membrane fractions after serum stimulation (Figure 2E). To confirm that IPMK-independent AKT membrane localization is not cell type-specific, we performed a cell fractionation study in the 786-0 cell line. After serum treatment, the transient knockdown of IPMK in 786-0 cells failed to impair AKT membrane localization but was markedly inhibited by pictilisib (Figures S2A and S2B). Thus, the PI3kinase activity of IPMK is essential for AKT Thr308 phosphorylation but dispensable for AKT membrane localization.

### PI3k activity of inositol polyphosphate multikinase mediates PDK1 membrane localization

**Figure 3:**
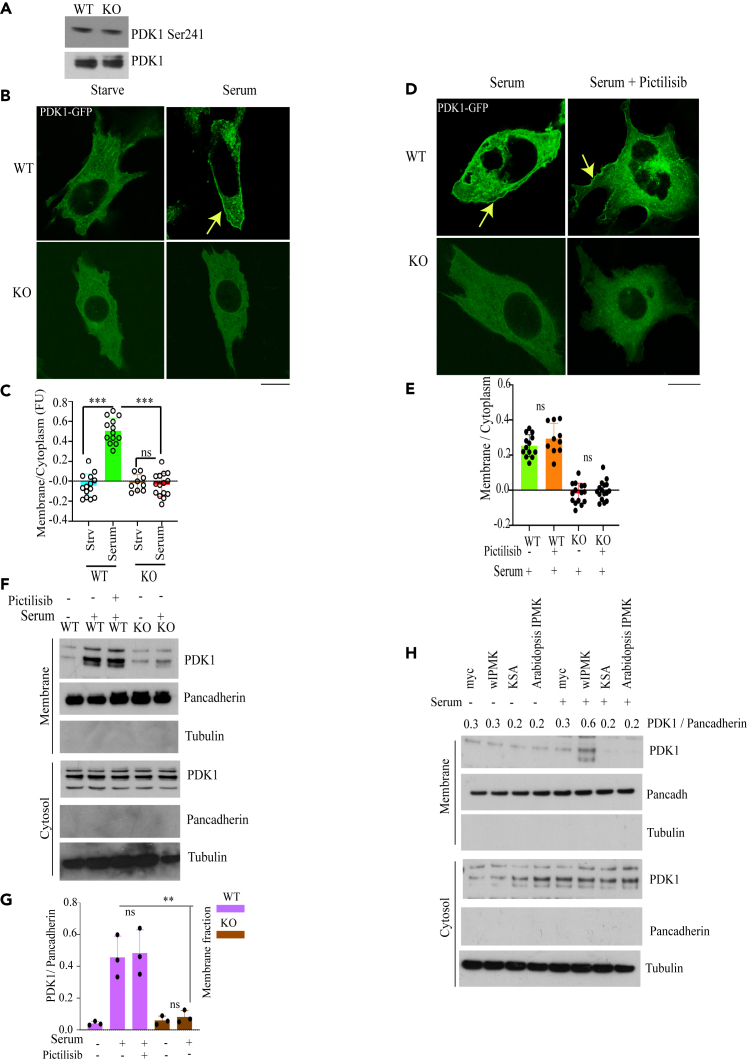
PI3k activity of IPMK mediates PDK1 membrane localization (A–E) Western blot of PDK1 Ser 241 in MEFs. Staining (B and D) (yellow arrow indicates membrane localized PDK1-GFP) and quantification (C is the quantification of B and E is the quantification of D) of overexpressed PDK1-GFP subcellular localization in IPMK WT and KO MEFs with or without serum stimulation and cPI3ki pictilisib. Membrane-localized PDK1-GFP is marked by the yellow arrow. Scale bar 20 μm. The bar graph shows the relative PDK1-GFP membrane/cytoplasm fluorescence ratio. ∗∗∗p < 0.001. Data are graphed as mean ± SD. (F and G) Western blot of endogenous PDK1 in cytoplasmic and membrane fractions isolated from WT and IPMK KO MEFs in response to serum stimulation. Pan-cadherin and tubulin blots confirm the purity of membrane and cytoplasm fractions, respectively. Data are graphed as mean ± SD. ∗∗p < 0.05, ns = not significant. (H) The cell fractionation study for PDK1 membrane localization for IPMK KO MEFs rescued with myc, wild-type IPMK, KSA, and *Arabidopsis* IPMK.

PDK1 is the essential AKT-kinase that phosphorylates AKT at Thr308.^8^ Growth factors stimulate PDK1 membrane recruitment and phosphorylation of AKT.^8^ We wondered whether IPMK directly stimulates the kinase activity of PDK1. PDK1 autophosphorylation at Ser 241 is necessary for PDK1's kinase activity.^18^ Depletion of IPMK in MEF failed to impair PDK1 Ser 241 phosphorylation (Figure 3A). To directly study the influence of IPMK on PDK1 kinase activity, we pulled down PDK1 from WT and KO MEFs and performed an *in vitro* kinase assay using AKT as a substrate. Deletion of IPMK did not impact PDK1-mediated AKT phosphorylation *in vitro* (Figure S3A). Thus, IPMK has no direct influence on PDK1 kinase activity.

Serum or growth factor stimulation promotes the generation of PIP_3, which binds to the PH domain of PDK1, translocating a fraction of PDK1 from the cytoplasm to the cell membrane.^10^ By contrast, inositol phosphate is not required for such action. At the membrane, PDK1 acts as an AKT kinase, phosphorylating AKT at Thr308.^8^

We hypothesized that IPMK-derived PIP_3 might influence PDK1 membrane localization. WT and KO MEFs were transiently transfected with full-length GFP-PDK1, followed by overnight serum starvation. As expected, treatment with serum induced the export of PDK1 to the cell membrane in WT MEFs, an action that was abolished in IPMK KO cells (Figures 3B and 3C). By contrast, pictilisib had only a marginal effect on PDK1 membrane localization (Figures 3D and 3E). We confirmed these microscopy data by western blotting of PDK1 from cytoplasmic and membrane fractions with or without serum stimulation (Figures 3F and 3G). Transient depletion of IPMK in 786-0 cells also inhibited PDK1 membrane localization, but was only slightly influenced by pictilisib (Figure S3B). The above data suggest that IPMK is the primary PI3k regulating PDK1 membrane localization.

To ascertain the importance of IPMK’s catalytic activity in regulating PDK1 membrane localization, we performed a western blot of PDK1 in cytoplasmic and membrane fractions from IPMK KO MEFs stably overexpressing empty vector (myc), WT IPMK, KSA or IPK2β. PDK1 membrane localization was evident in cells rescued with WT IPMK but was absent in cells rescued with either empty vector, KSA or IPK2β (*Arabidopsis* IPMK) (Figure 3H), confirming that the lipid kinase activity of IPMK is required for PDK1 membrane localization.

PDK1 membrane localization is also known to impair ROCK1/RhoE binding, resulting in ROCK1 activation, phosphorylation of myosin light chain (MLC), and cell migration.^12^^,^^13^ Previous studies^12^ showed that PIP_3 promotes the localization of PDK1 to the cell membrane, where it dissociates the ROCK1-inhibiting RhoE from ROCK1, with effects only modestly impacted by cPI3ki. We examined the influence of IPMK upon ROCK1/RhoE binding by the immunoprecipitation of ROCK1. RhoE/ROCK1 interactions were equal under serum starvation conditions in WT and KO MEFs. Stimulation with serum for 5 min markedly depleted ROCK1 binding to RhoE in WT cells, whereas KO cells maintained the ROCK1/RhoE complex at levels comparable to serum starvation (Figure S3C). Myosin light chain (MLC) is a downstream substrate of ROCK1,^13^ and phosphorylation MLC promotes cell migration by modulating actin polymerization.^12^^,^^13^ Deletion of IPMK markedly reduced MLC phosphorylation (Figure S3D) and cell migration (Figure S3E). These data further confirm that IPMK promotes PDK1 membrane localization and its downstream signaling events.

### Inositol polyphosphate multikinase is essential for intestinal integrity

Previous RNA *in situ* hybridization studies established high IPMK expression in the intestinal ileum.^6^

**Figure 4:**
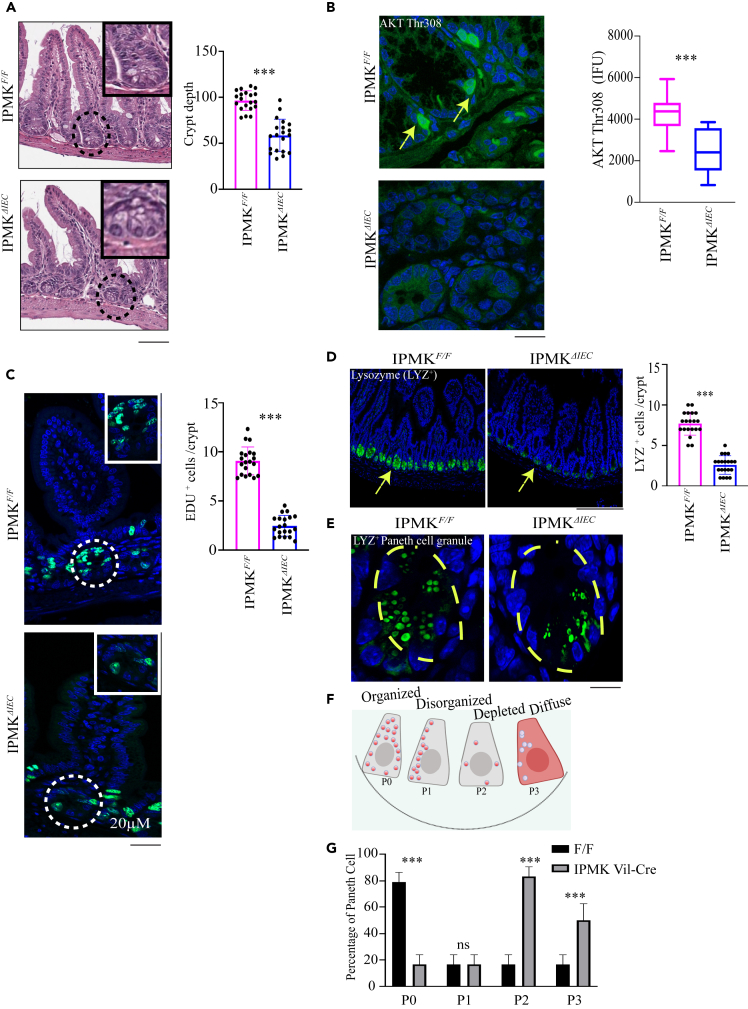
IPMK is essential for intestinal integrity (A) H&E staining of ileum sections from *Ipmk*^*F/F*^ and *Ipmk*^*ΔIEC*^ mice. The graph depicts crypt depth. n = 4 mice each group, ∗∗∗p < 0.001. The black circle is the crypt shown in a magnified view (black square). Data are graphed as mean ± SD. Scale bar 200 μm. (B) AKT Thr308 staining (green) of ileum sections from *Ipmk*^*F/F*^ and *Ipmk*^*ΔIEC*^ mice, n = 4 mice in each group. ∗∗∗p < 0.001. Data are graphed as mean ± SD. DAPI (blue) was used for nuclear staining. The yellow arrow indicates AKT Thr308 staining. Scale bar 10 μm. (C) EdU (green) staining in intestinal sections from *Ipmk*^*F/F*^ and *Ipmk*^*ΔIEC*^ mice intraperitoneally injected with EdU 2h prior to sacrifice. DAPI (blue) was used for nuclear staining. The white circle is the crypt shown in a magnified view (white square). The number of EdU-positive cells were counted, n = 4 mice in each group. ∗∗∗p < 0.001. Data are graphed as mean ± SD. Scale bar 20 μm. (D and E) Paneth cell granules were identified by lysozyme (LYZ) staining (green) in ileum sections from *Ipmk*^*F/F*^ and *Ipmk*^*ΔIEC*^ mice, n = 4 mice in each group. ∗∗∗p < 0.001. (D) is the low magnification (20× air) image scale bar 50 μm. The yellow arrow indicates LYZ positive crypts, and (E) is a high magnification (60× oil) image scale bar 10 μm. The yellow circle shows the Paneth cell in the side crypt. Data are graphed as mean ± SD. DAPI (blue) was used for nuclear staining. (F and G) Cartoon (F) and quantification (G) of lysosomal granule distribution in Paneth cells in *Ipmk*^*F/F*^ and *Ipmk*^*ΔIEC*^ ileum sections. n = 4 mice in each group. ∗∗∗p < 0.001. Data are graphed as mean ± SD.

To investigate a potential role for IPMK in the activation of AKT and intestinal homeostasis, we crossed *Ipmk* floxed mice (*Ipmk*^*F/F*^) with *Villin-Cre* mice to generate a mouse line lacking *Ipmk* selectively in intestinal epithelial cells (IECs) (*Ipmk*^*ΔIEC*^) (Figure S1F). *Ipmk*^*ΔIEC*^ mice were born at the expected Mendelian ratios and developed normally. IEC-specific deletion of *Ipmk* in 8 to 12-weeks old mice resulted in decreased crypt depth in the ileum (Figure 4A). Cell proliferation in the intestinal epithelium, one of the most proliferative tissues in mammals, requires the activation of AKT.^19^ *Ipmk*^*F/F*^ mice showed prominent AKT Thr308 staining, which was markedly diminished in *Ipmk*^*ΔIEC*^ mice (Figure 4B), demonstrating that IPMK is a physiological regulator of AKT *in vivo*.

Next, we examined the importance of IPMK in IEC proliferation under basal conditions. *Ipmk*-deleted IECs showed a marked loss of incorporation of 5-ethynyl-2′-deoxyuridine (EdU), a thymidine analog that is incorporated into the DNA of proliferative cells, suggesting that IPMK is critical for IEC proliferation (Figure 4C). To further examine the effect of IPMK deletion on intestinal proliferation, we cultured enteroids isolated from *Ipmk*^*F/F*^ and *Ipmk*^*ΔIEC*^ small intestines. After 7 days of culture, enteroids from *Ipmk*^*F/F*^ mice showed proper budded structure, while *Ipmk*^*ΔIEC*^ enteroids showed less morphological complexity (Figures S4A and S4B). Culture of an equal initial number of cells from *Ipmk*^*ΔIEC*^ mice gave rise to fewer enteroids than cells cultured from *Ipmk*^*F/F*^ mice (Figure S4B). To ascertain the effect of AKT and PDK1 on the proliferation of enteroids, we incubated *Ipmk*^*F/F*^ enteroids for 7 days with specific AKT and PDK1 inhibitors. AKT and PDK1 inhibitors both phenocopied IPMK deletion, reducing the enteroid abundance and producing enteroids with deformed structure (Figures S4A and S4B)*.* AKT is a master regulator of cell proliferation and can play this role through multiple mechanisms. In IECs, phosphorylation of β-catenin at Ser552 by AKT elicits its translocation to the nucleus, where β-catenin acts as a transcriptional coactivator to stimulate cellular proliferation.^20^ Nuclear localization of β-catenin Ser 522 was notably reduced in *Ipmk*^*ΔIEC*^ mice at the intestinal crypt base (Figure S4C), which was further validated by western blot from enteroid (*Ipmk*^*F/F*^*/Ipmk*^*ΔIEC*^*)* lysates (Figure S4C). Wnt 3A signaling upstream of β-catenin is directly related to intestinal regeneration. We wondered about the influence of IPMK for Wnt 3A dependent gene expressions. Axin2, c-Myc, and Lef1 are important Wnt3A-dependent genes critical for intestinal homeostasis.^21^ Interestingly IPMK depletion considerably impaired Axin2 protein expression while c-Myc and Lef1 expression in IPMK KO MEF cells were comparable to the WT (Figure S4D). Axin 2 protein expression in *Ipmk*^*ΔIEC*^ enteroids was also reduced compared to *Ipmk*^*F/F*^ confirming the cell line data (Figure S4E). This indicates that IPMK is critical for Wnt 3A-mediated Axin2 expression; however, a more in-depth examination of the significance of IPMK on canonical Wnt signaling activation in intestinal regeneration will be conducted in a future study.

Activation of AKT is essential for the homeostasis of Paneth cells, specialized crypt-resident cells that support intestinal regeneration by maintaining intestinal stem cell homeostasis.^22^ Intestinal deletion of IPMK elicited a 50% decrease in Paneth cell number (Figure 4D). Higher magnification confocal imaging of Paneth cell marker Lysozyme (Figures 4E–4G) and Periodic acid-Schiff (PAS)/alcian-blue-staining (Figure S4F) of intestinal sections of *Ipmk*^*ΔIEC*^ mice, revealed pronounced abnormalities in Paneth cells, including aberrant, disorganized granules, as well as decreased numbers of granules (Figures 4E–4G and S4F). Histological analysis of these sections from four controls and four mutant mice revealed a 100% concordance between *Ipmk* genotype and abnormal Paneth cell morphology.

Paneth cell disorganization is known to contribute to Crohn disease pathology.^23^ GWAS data^7^ revealed a significant association between *IPMK* variants and risk for IBD. Four SNPs at the *IPMK* locus were significantly associated with IBD. SNPs that have statistically significant associations with Crohn disease include rs1819658, located in an enhancer of the *IPMK* gene (p = 9.0 x 10^−17^), and rs2790216, located in intron 1 of *IPMK* (p = 8.0 x 10^−9^) (Figure S4G). Two additional SNPs were also associated with IBD (not presented in Figure S4G): rs2153283, located in intron 4 of *IPMK* (IBD p = 2 x 10^−11^, MAF: 0.40, alleles C/A), and rs1199103, an intergenic variant (IBD, p = 5 x 10^−11^, MAF: 0.40, alleles A/C/G).

Epithelial cells are born at the base of the intestinal crypts and migrate to the tip of the villus, where they reside until they die (Figure S4H). A recent study showed that active cell migration is critical for maintaining intestinal homeostasis.^23^ We examined the influence of IPMK on IEC migration 48 h after injection of EdU. Intestinal deletion of IPMK strikingly impeded IEC migration from the crypt to the villus tip (Figure S4H). In summary, loss of IPMK expression in IECs led to impaired epithelial cell proliferation, Paneth cell abnormalities, and diminution of IEC migration to the villus tip.

### Inositol polyphosphate multikinase enhances intestinal epithelial repair

**Figure 5:**
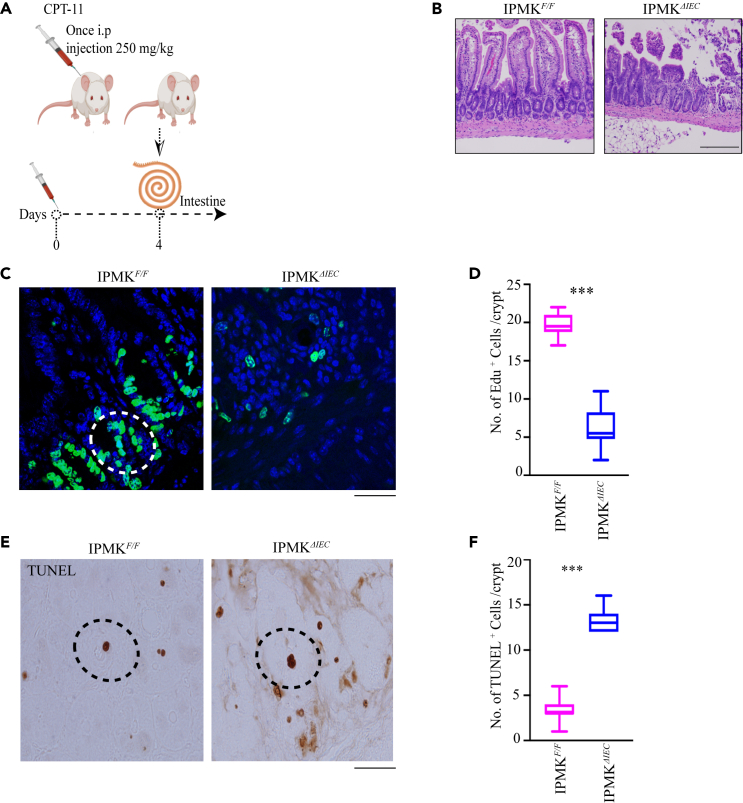
IPMK enhances intestinal epithelial repair (A) Schematic of CPT-11 treatment plan. (B) H&E staining of CPT-11-treated ileum sections from *Ipmk*^*ΔIEC*^ and *Ipmk*^F/F^ mice. Scale bar 200 μm. (C) EdU (green) and DAPI (blue) staining 2h after injection in CPT-11-treated intestines. Scale bar 20 μm (white circle specifies Edu positive cell). (D) Quantitation of EdU-positive cells. n = 4 mice each group, ∗∗∗p < 0.001. Data are graphed as mean ± SD. (E and F) TUNEL-positive cells (dark brown and black dotted circle) after CPT-11 treatment in *Ipmk*^*F/F*^ and *Ipmk*^*ΔIEC*^ ileum. Scale bar 20 μm, (F) Quantitation of TUNEL-positive cells. n = 4 mice each group, ∗∗∗p < 0.001. Data are graphed as mean ± SD.

AKT promotes IEC proliferation during damage-induced intestinal tissue repair.^22^ To investigate a potential role for IPMK in recovery from chemotherapy-induced acute intestinal injury, we treated *Ipmk*^*F/F*^ and *Ipmk*^*ΔIEC*^ mice with a single dose of CPT-11 (irinotecan) (250 mg/kg), an agent currently being explored as a treatment for colorectal cancer (Figure 5A) and analyzed intestinal tissue 4 days later. Histopathological analysis revealed the regeneration of crypts and epithelial layers in *Ipmk*^*F/F*^ mice. By contrast, *Ipmk*^*ΔIEC*^ mice displayed significant impairment in the healing of crypts (Figure 5B). We found extensive depletion of EdU-positive cells in *Ipmk*^*ΔIEC*^ mice relative to *Ipmk*^*F/F*^ mice, indicating that IPMK is a mediator of intestinal regeneration (Figures 5C and 5D). Loss of regeneration in *Ipmk*^*ΔIEC*^ mice was associated with a pronounced increase in apoptosis, as evident from TUNEL staining (Figures 5E and 5F). Taken together, these data demonstrate that IPMK plays a critical role in regenerative responses to intestinal toxins.

## Discussion

We previously established IPMK as a novel mammalian PI3k.^3^ Recently, Jung et al. showed loss of AKT activation in IPMK-depleted primary hepatocytes.^24^ However, the physiological implications of IPMK’s PI3k activity have remained obscure. Here we show that the PI3k activity of IPMK is critical for PDK1 membrane localization, which promotes the activation of AKT and ROCK1 to stimulate intestinal epithelial cell migration, proliferation, and regeneration.

It is well accepted that cPI3k generates PIP_3 at the plasma membrane, an action required for AKT membrane localization and PDK1- and mTORC2-mediated AKT phosphorylation. We found that the deletion of IPMK abolished AKT phosphorylation at both Thr308 and Ser473 by around 60% (Figures 1D–1F, S1D, and S1E), a deficit which could be rescued by WT IPMK, but not by an IPMK mutant lacking kinase activity (Figure 3H). However, the PI3k activity of cPI3k (p110 α,β,γ,andδ) remained intact after IPMK deletion (Figure S1C). Accordingly, we explored mechanisms of IPMK-mediated AKT activation. Confocal imaging and cell fractionation studies demonstrated that AKT membrane localization was independent of IPMK and mediated by cPI3k (Figures 2C–2E). Previously, we showed^5^ that the deletion of IPMK impaired mTORC2 activation and thus diminished AKT Ser473 phosphorylation (Figures S1D and S1E). We wondered whether IPMK-derived PIP_3 controls the kinases that phosphorylate AKT. PDK1 is the first discovered AKT kinase and, like AKT itself, is recruited to the membrane by PIP_3.^16^ The cPI3ki wortmannin^22^ fails to inhibit PDK1 membrane localization.^10^ Similarly, *Pinner* et al.^12^ found that PIP_3-mediated PDK1 membrane localization helps to dissociate ROCK1 from inhibitory RhoE binding, thus promoting cell migration; this action was likewise mostly unaffected by cPI3ki.^12^ We also showed that a cPI3ki had no effect on the PI3k activity of IPMK.^3^ Here we found that the PI3k activity of IPMK is critical for PDK1 membrane localization, which was minimally influenced by the cPI3ki pictilisib (Figures 3B–3F). Deletion of IPMK also impaired PDK1-mediated ROCK1 activation by preventing the dissociation of ROCK1 from RhoE (Figures S3C and S3D) and diminished cell migration (Figure S3E), which is in agreement with the previous study by Sekine et al.^25^ However, the recruitment of PDK1 to the membrane does not require physical interaction between PDK1 and IPMK (data not shown). This finding establishes IPMK as a critical mammalian PI3k that promotes the membrane localization of PDK1, one of the principal kinases that triggers AKT activation. In contrast to conventional wisdom, PDK1 membrane localization is mostly independent of cPI3k.

Activation of AKT is essential for maintaining the homeostasis of regenerative organs.^24^ The intestine is a highly regenerative organ, and the loss of AKT function in the intestine impairs intestinal epithelial cell regeneration.^22^ Activation of AKT is critical for maintaining homeostasis of the Paneth cell, a specialized intestinal cell type that resides in ileal crypts and secretes antibacterial peptides and growth factors to regulate intestinal bacterial flora and nurture intestinal stem cells. Intestinal deletion of IPMK markedly reduced PDK1-mediated AKT Thr308 phosphorylation (Figure 4B) and IEC proliferation both under basal conditions and during recovery from chemotherapy-induced tissue damage (Figures 4C and 5). *Ipmk*^*ΔIEC*^ mice showed a striking loss of Paneth cell number accompanied by pronounced abnormalities in the organization of Paneth cell granules (Figures 4D–4G). This Paneth cell abnormality has been observed in Crohn disease.^26^ Genome-wide association studies previously identified IPMK as a major Crohn’s disease-related gene (Figure S4G). Thus, loss of function of IPMK may drive pathology in Crohn disease and IBD by impairing AKT-mediated Paneth cell integrity. Detailed analysis of how IPMK influences Paneth cell biology and maintenance of ISC (intestinal stem cell) will be done in future studies creating Paneth cell-specific and ISC-specific IPMK KO mice lines.

**Figure 6:**
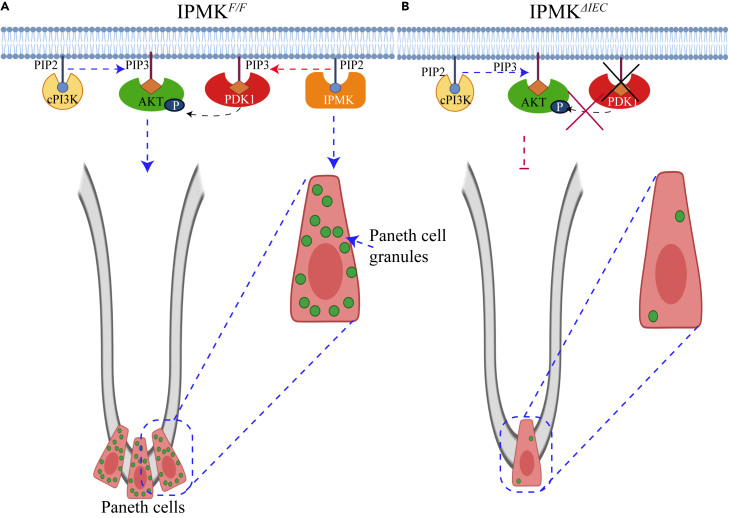
Schematic diagram depicting the role of IPMK in PDK1-mediated AKT activation and intestinal homeostasis (A) Under normal conditions, IPMK promotes PDK1 membrane localization and PDK1-dependent AKT phosphorylation. The PI3k activity of IPMK is, however, expendable for AKT membrane localization, which exclusively depends on cPI3k. PDK1-dependent activation of AKT maintains Paneth cell homeostasis. (B) Deletion of IPMK (*Ipmk*^*ΔIEC*^) impairs PDK1 membrane localization and PDK1-dependent AKT phosphorylation, rendering AKT inactive. Loss of function of AKT impairs intestinal proliferation and Paneth cell function in the crypt (e.g., loss of Paneth cell number and dysregulation of intracellular granules).

In summary, IPMK is a novel PI3k for PDK1 that activates AKT and promotes ROCK1-mediated cell migration. PDK1 membrane localization and PDK1-dependent AKT phosphorylation are critically regulated by the PI3k activity of IPMK and are independent of cPI3k. By contrast, the PI3k activity of IPMK is dispensable for AKT membrane localization, which depends exclusively on cPI3k (Figure 6A). IPMK-mediated AKT activation promotes intestinal regeneration by maintaining Paneth cell homeostasis. Differences in IPMK activity may contribute to the risk for Crohn disease, and drugs targeting the PI3k activity of IPMK may have potential as novel treatments for Crohn disease and related conditions.

### Limitations of the study

Intestinal regeneration is directly linked to several intestinal diseases. Crosstalks of different cell types with intestinal stem cells determine the fate of intestinal regeneration. Our research, for the first time, shows the importance of IPMK in ileum health. In our study, the observations of loss of Paneth cells in IPMK-deleted intestine indicates that IPMK is critical for intestinal regeneration and may be linked to Crohn disease. While our study has uncovered roles of IPMK in intestinal regeneration, it is predominantly observational and missing a direct link with intestinal stem cells. Future work will help understand the link between IPMK/intestinal stem cell and regeneration.

## STAR★Methods

### Key resources table

**Table:** 

REAGENT or RESOURCE	SOURCE	IDENTIFIER
**Antibodies**		

β-Actin	Cell Signaling	4967;RRID: AB_330288
phospho-myosin light chain 2 (Thr18/Ser19)	Cell Signaling	3674
RhoE	Cell Signaling	3664
phospho-AKT (Ser473)	Cell Signaling	4060
phospho-AKT (Thr308)	Gene Tex Cell Signaling	GTX79150 9275
Lysozyme	Ab Cam	108508
Phospho-β-catenin-S552	ABclonal	AP0579
Myosin Light Chain 2 antibody	Proteintech	AP0579
ROCK1	Bethyl Laboratories	A300-457A
Goat anti-Rabbit, Alexa Fluor 488 antibody	Invitrogen	A-11008
GSK2α Ser 21	Cell Signaling	9331
HRP-Anti Mouse	GE Health care	NA931V
IgG control antibody	Cell signaling	2729
Mouse specific Anti-IPMK	Lab generated	NA
HRP-Anti Rabbit	GE Health care	GENA934
GFP	Cell Signaling	2555
Myc	Cell Signaling	2276

**Biological samples**		

AKT-PH-GFP	Addgene	51465
GFP-PDK1	Addgene	134189
pMX-myc	Snyder Lab	NA
pMX-wIPMK	Snyder Lab	NA
pMX-IPMK-KSA	Snyder Lab	NA

**Chemicals, peptides and recombinant proteins**		

Pictilisib GDC-0941	Millipore Sigma	957054-30-7
LY294002	Selleckchem	S1105
Ezview myc Beads	Sigma	E6654
PVDF membrane	Millipore Sigma	IPVH00010
DMEM (w/o glucose)	Thermo Fischer Scientific	25030081
FBS	Thermo Fischer Scientific	F2442
DMEM	Thermo Fischer Scientific	11960-0; 512449655
L-glutamine	Thermo Fischer Scientific	25030081
Lipofectamine 3000	Thermo Fischer Scientific	L3000015
Paraformaldehyde 4% solution in PBS	Thermo Fischer Scientific	J19943.K2
Hydrogen peroxide	Thermo Fischer Scientific	202465000
Penicillin/streptomycin	Thermo Fischer Scientific	15140122
DMSO Cell culture grade	Thermo Fischer Scientific	J66650.AP
Irinotecan hydrochloride (CPT-11)	Sigma	I1406-50mg
ImmPACT® DAB Peroxidase (HRP) Substrate	Vector Biolab	SK-4105
DyLight 594 Streptavidin	Vector Biolab	SA-5594
Antigen Retrieval Reagent-Universal	R&D systems	CTS015
EZview™ Red Protein G Affinity Gel	Sigma-Aldrich	E3403-1ml
Click-iT™ EdU Cell Proliferation Kit for Imaging, Alexa Fluor™ 488	ThermoFisher	C10337
VECTASHIELD® Antifade Mounting Medium	Vector Biolab	H-1000-10
2.5% Normal Goat Serum Blocking Solution	Vector Biolab	S-1012-50
Citrate Buffer pH 6.0	Sigma Aldrich	C9999-100ml
Polybrene	Tocris	7711
Blasticidin	Thermo Scientific	A1113903
HEPES	Sigma Life science	H0887
Potassium acetate	Thermo Scientific	AM9610
MgCl2	Thermo Scientific	012315.C4
Recombinant AKT1/PKB1 protein	Millipore Sigma	14-279
Super Signal ^TM^ West Femto Luminol	Thermo Scientific	1859022
Recombinant Wnt 3A	R&D system	5036-WN
Histo-Clear	Fisher Scientific	5089990147
6-diamidino-2-phenylindole	Thermo Fisher Scientific	D1306
IntestiCult™ Enteroid Growth Medium	STEM CELL Technologies	06005
Gentle Cell Dissociation Reagent	STEM CELL Technologies	100-0485

**Critical commercial assays**		

PIP_3 mass ELISA kit	Echelon Biosciences	K-2500S
ProteoExtract® Subcellular Proteome Extraction Kit	Millipore-Sigma	539790
TumorTACS™ *In Situ* Apoptosis Detection Kit	R&D Systems	4815-30-K
Periodic Acid Schiff Stain Kit	Abcam	Ab150680

**Experimental models: Cell lines**		

Mouse Embryonic fibroblast	Snyder Lab	NA
Human Embryonic Kidney Cell	ATCC	ATCC-CRL-1932

**Experimental models: Organisms/strains**		

IPMK F/F mice 129SV-C57 BL/6 mixed background	Ozgene	Custom Developed
Albumin Cre mice c57BL/6J	Jackson laboratory	3574
*Ipmk*^*ΔIEC*^	Ozgene	Custom Developer

**Software and algorithms**		

ZEN lite	Carl Zeiss	https://www.zeiss.com/microscopy/int/downloads.html?vaURL=www.zeiss.com/microscopy/int/downloads/zen.html
ImageJ	NIH ImageJ	https://imagej.nih.gov/ij/
Graph Pad prism 8	Graph Pad prism	https://www.graphpad.com/scientific-software/prism/
Illustrator CC	Adobe	https://www.adobe.com/products/illustrator/free-trial-download.html
ImageXpress Micro XLS widefield high-content analysis system	Molecular devices	https://www.moleculardevices.com/products/cellular-imaging-systems?
ImarisX64	OXFORD instruments	https://imaris.oxinst.com/products/imaris-for-cell-biologists?gclid=CjwKCAiAjPyfBhBMEiwAB2CCIt-pe1x8x9GzTbItQo2bRnx74Tdcycz-zEQD5DxMwi6zZbmcvOZv6RoCwgIQAvD_BwE

### Resource availability

#### Lead contact

Further information and requests for resources and reagents should be fulfilled by the Lead Contact, Dr. Prasun Guha, Ph.D. (prasun.guha@unlv.edu).

#### Materials availability

Plasmids and mouse lines generated in this study are available from the lead contact upon reasonable request with a completed Materials Transfer Agreement.

### Experimental model and subject details

#### Animals

All protocols were approved by the Johns Hopkins University Animal Care and Use Committee. Mice were housed according to institutional guidelines, in a controlled environment at a temperature of 22_C ± 1_C, under a 12-h dark-light period and provided with standard chow diet and water *ad libitum*. Male and female IPMK F/F, IPMK F/F-Alb Cre, (between 1 and 3-month-old) were used. Specifically, male mice were used for liver regeneration study, electron microscopy and tissue histology. All mice were maintained in 129SV-C57BL/6 mixed background.

#### Generation of intestine-specific IPMK KO mice

*Ipmk*^*F/F*^ mice were generated as previously described (2*2). Ipmk*^*F/F*^ mice were crossed with C57BL/6J mice carrying Cre expressed under the control of the murine villin promoter as described previously (34) to create intestinal-specific *Ipmk* knockout mice (*Ipmk*^*ΔIEC*^). Homozygous *Ipmk*^*F/F*^ mice were crossed with the *Ipmk*^*ΔIEC*^ mice, which mediate excision of floxed alleles in the intestine. All mice were maintained on a C57BL/6J background and were 7th generation backcrossed. C57BL/6J mice were used for Enteroid isolation for inhibitor treatment as well as for DSS and CPT-11 treatments. Mice were housed in a 12-hour light/12-hour dark cycle at 22°C and fed standard rodent chow. All research involving mice was approved by the Johns Hopkins Animal Care and Use Committee.

#### Cell culture

Mouse embryonic fibroblast (MEF) and human embryonic kidney (HEK) cells were cultured in Dulbecco's modified Eagles medium (DMEM) supplemented with 10% FBS, 2 mM L-glutamine, 100 U/mL penicillin, and 100 mg/mL streptomycin. IPMK WT and KO MEF cells were developed in our lab as previously described,^3^ briefly, floxed MEFs were immortalized by transfection with pSG5- Large T (deposited by William Hahn to Addgene) using Polyfect transfection reagent (Qiagen) according to the manufacturer's protocol. After serial-passaging transfected cells five times to select against nontransformed cells, IPMK KO cells were generated by transduction of floxed cells with adenovirus carrying the gene encoding the Cre recombinase (University of Iowa Gene Transfer Vector Core). The virus was combined with GeneJammer (Stratagene) transfection reagent to enhance transduction efficiency. After transduction, clonal populations were colony purified and screened for IPMK expression.^3^

#### Plasmids and transfection

AKT-PH-GFP and GFP-PDK1 plasmids were from Addgene. pMX-myc, pMX-wIPMK and pMX-IPMK-KSA were generated in-lab.

#### Retroviral transfection and generation of stably transfected cells

Target vector DNA retroviral constructs were transiently transfected into a Platinum-E (Plat-E) retrovirus packaging cell line for 48 hours by using Lipofectamine 3000 transfection reagent. High-titer viral stocks were harvested by passing the supernatant through a 0.45 μm filter. For infection, MEFs were incubated with the viral supernatant in the presence of polybrene (8μg/mL) for 48 hours. Stably infected MEFs were selected by treatment with blasticidin (4 μg/mL) for 1-2 weeks. Selected stable cell lines were continuously maintained in medium containing blasticidin (4 μg/mL).

#### Transient transfection

MEF cells were transfected with Lipofectamine 3000 (ThermoFisher) per manufacturer protocol.

#### AKT activation in cell culture

To evaluate AKT phosphorylation in MEF cells, 5x10^6^ cells were seeded in 10 cm plates and left to attach to the plate for 3 hours. Cells were then washed with room temperature phosphate-buffered saline (PBS) followed by incubation of cells in serum-free DMEM supplemented with 2 mM L-glutamine, 100 U/mL penicillin, and 100 mg/mL streptomycin. After 24 hours of serum starvation, serum-free media was replaced with complete DMEM (10% FBS) for 5 minutes to promote AKT membrane localization. Cells given the cPI3ki GDC0941/pictilisib were pretreated for 30 minutes prior to complete DMEM administration with GDC0941 (100 nM) in serum-free media. After 5 minutes of serum stimulation, cells were quenched with cold PBS and lysed as previously described.

#### Cytoplasm and cell membrane isolation

Cytoplasm and membrane fractions were obtained using the ProteoExtract® Subcellular Proteome Extraction Kit (Millipore-Sigma] following manufacturer protocol.

#### Immunoprecipitation

Immunoprecipitation of endogenous ROCK-I was performed with 1 mg of protein lysates in lysis buffer (150mMNaCl, 0.5% CHAPS, 0.1% Triton, 0.1% BSA, 1mM EDTA, protease inhibitors, and phosphatase inhibitors). Protein lysate was incubated for 2 hrs. at 4∗C with ROCK1 antibody (Bethyl) followed by EZview Protein G beads (Sigma) for 1 h. Beads were pelleted at 1000xg and washed with lysis buffer 3 times for 5 minutes each on a rocking platform at 4∗C, and SDS sample buffer loading dye was added. Immunoprecipitated samples were resolved by polyacrylamide gel electrophoresis and binding of RhoE was observed by Western blot.

#### *In vitro* AKT and PDK1 kinase activity assay

To perform *In vitro* PDK1 kinase assay 3 μg of PDK1/ AKT antibody was added to 1.5 mg of lysates and incubated with rotation for 2 hours at 4°C. 15 μL of protein G beads was added and the incubation continued for an additional hour. PDK1/AKT immunoprecipitates were washed four times with the same lysis buffer and twice with the kinase buffer (25 mM HEPES [pH 7.5], 100 mM potassium acetate, 1 mM MgCl2). Kinase assays were performed for 20 min at 37 °C in a final volume of 15 microliters of kinase buffer containing 500 μM ATP and 500 ng inactive AKT1/PKB1 (Millipore) as a substrate for PDK1 and GSK3 α for AKT. Reaction was stopped by resuspending beads in SDS-containing sample buffer and boiling samples for 5 minutes followed by western blot of phosphor-AKT Thr308 and GSK3 α Ser 21.

#### AKT and PDK1 membrane localization

5x10^5^ MEF cells were seeded on 35 mm glass-bottom cell culture dishes and left to proliferate for 3 hours. Cells were then transfected with 500 ng AKT-PH-GFP using Lipofectamine 3000 following manufacturer's recommendations. 24 hours after transfection, cells were washed with PBS and deprived of serum by exchanging complete DMEM with FBS-free media as previously described. After 24 hours of serum starvation, serum-free media was replaced with complete DMEM (10% FBS). Cells given the cPI3ki GDC0941/pictilisib were treated as described above. After 5 minutes of serum stimulation, cells were quenched with cold PBS and fixed using chilled 4% paraformaldehyde. Cells were stained with DAPI (1 μg/mL) in PBS. After staining, coverslips were mounted with antifade (Vectashield) mounting media. Cells were imaged via confocal microscopy; images were exported as .czi files to ImageJ (NIH). For each image, well-defined and intact portions of the membrane were traced using the “segmented line” function with spline-fit, setting the line thickness such that the selection covered 2.5 μm across the membrane. This was followed by transformation using the "straighten" function to generate a uniform, straight section of membrane. This image was rotated such that the left of the image was the intracellular space and the right side was the extracellular space, with the edge of the membrane lying in the middle of the image. A plot profile was generated using this image, yielding a trace of the gray value across the membrane, progressing intra- to extracellular down the x-axis. The data produced by this plot was exported to Excel. As the transfection efficiency differed slightly between cells, data from each image was normalized from 0 to 1, with 0 being the extracellular space and 1 being the maximum gray value. Using this data, the difference between cytoplasm and membrane fluorescence was calculated.

#### Western Blot

Cell lysates were first prepared using lysis buffer (50mM Tris-HCl, 150mM NaCl, 1mM EDTA, 1% Triton X-100 in PBS, phosphatase inhibitors, protease inhibitors). Samples were centrifuged at 16,200xg for 10 min, followed by total protein quantification. Proteins were resolved by SDS-polyacrylamide gel electrophoresis and transferred to PVDF membranes. Membranes were incubated in 1:1000 dilution of primary antibody in 20 mM Tris-HCl (pH 7.4), 150 mM NaCl, and 0.02% Tween 20 (Tris-buffered saline/Tween 20, TBST) with 3% BSA overnight at 4°C on a rocking platform. The membranes were washed 3 times with Tris-buffered saline/Tween-20, then incubated with HRP-conjugated secondary antibody (GE Health Care) diluted 1:5000 in TBST with 3% BSA, and the bands visualized by chemiluminescence (ThermoFisher). The blots shown in the figures were representative replicates selected from at least 3 experiments. Densitometric analysis was performed using ImageJ software.

#### Wnt 3A treatment and protein expression

Recombinant Wnt 3A (R&D system) was diluted in water. Cells were treated with 250 ng/ml of Wnt 3A diluted in serum-free medium and incubated for 24h.

#### Immunohistochemistry, immunofluorescence and DAB stain

Mice were sacrificed via CO_2_. Immediately following euthanasia, the intestine was removed and flushed with cold PBS. The intestine was subsequently opened lengthwise and washed in cold PBS, then cut into approximately 3-4 mm fragments for sectioning and paraffinization. Samples were deparaffinized with Histo-Clear (Thermo Fisher) and rehydrated in successive washes of 100%, 90%, and 75% ethanol followed by deionized (DI) water. Sections were unmasked via heat-induced epitope retrieval (HIER) using citrate buffer, followed by blocking with 2.5% goat serum for 1 hour. Primary antibodies were diluted per manufacturer's recommendation in Triton-X diluted to 1% in PBS, incubating overnight at 4°C in a humidified chamber. The following day, samples were washed with TBST, followed by incubation with fluorescent-tagged secondary antibodies diluted in 1% Triton-X solution for 60 minutes. After washing with TBST, samples were stained with 4′, 6-diamidino-2-phenylindole (DAPI) diluted in PBS (1 μg/mL). Slides were imaged using a slide scanner and confocal microscopy.

To identify the influence of IPMK deletion on Paneth cell, we used lysozyme to detect Paneth cells. These, along with anti-Beta-catenin Ser 522, anti-IPMK, and IgG control antibodies, were all anti-rabbit and targeted with fluorescent secondary Alexa Fluor488. Additionally, F4/80 staining (used to detect inflammatory cells) was detected using Alexa Fluor 488-tagged anti-mouse secondary antibodies. Phospho-AKT Thr308 antibodies were targeted with Alexa Fluor488-tagged anti-rabbit antibodies.

Tissue samples of the small intestine, large intestine, lung, kidney, liver, skeletal muscle, cardiac muscle, pancreas, skin, and lymph were sectioned and paraffinized as described above. Endogenous peroxidase activity was first blocked with [0.1%] H_2_O_2_ for 5 minutes. Sections were incubated with IPMK antibody followed by HRP-tagged secondary antibody, then treated with diaminobenzidine (DAB) substrate. Sections were counterstained with hematoxylin. Images were acquired at 40x magnification with an Olympus microscope. Hematoxylin and DAB channels were separated using the ImageJ color deconvolution macro, which utilizes an RGB image deconvolution method developed by Ruifrok and Johnston (36). Subsequent histogram analysis in ImageJ produced signal intensities from 0-255, with 0 = darkest intensity (positive staining) and 255 representing a white, unstained signal. A threshold of 0-230 was applied to eliminate background staining; using this threshold, the MGV of the tissue samples was determined. Using the MGV, the optical density (OD) of each piece was determined using the equation $OD=\log_{10}\frac{I_{0}}{I_{1}}$, where $I_{0}$ is the intensity of transmitted light (255) and $I_{1}$ is the intensity of the DAB-positive area of the sample.

#### TUNEL staining

Histologic sections were stained using TumorTACS™ *In Situ* Apoptosis Detection Kit (Trevigen) following manufacturer protocol.

#### Periodic acid Schiff staining

Samples were first deparaffinized with Histo-Clear and rehydrated in successive washes of 100%, 90%, and 75% ethanol, followed by a wash in deionized (DI) water. Slides were stained using the Periodic Acid Schiff Stain Kit (Abcam) following the manufacturer's protocol.

#### EdU treatment and tissue regeneration study

To evaluate DNA synthesis as a marker for epithelial regeneration, mice were given a single administration of 5–ethynyl–2′–deoxyuridine (EdU) by intraperitoneal injection at [200ug/kg] in PBS. Mice were sacrificed 2 hours after administration, with both small and large intestines prepared for histological analysis using Click-iT™ EdU Cell Proliferation Kit for Imaging, Alexa Fluor™ 488 (Thermo Fisher) according to manufacturer protocol.

#### Intestinal and MEF cell migration

To check cell migration in MEF, we performed a scratch wound assay. WT and KO MEFs were plated in a glass bottom tissue culture plate with a silicon spacer in the middle. Once cells adhered they were kept in a serum-starved medium overnight. The next day, the serum free medium was replaced by plus serum medium, and the spacer was gently removed. After 7 h, the cell migration was captured under a light microscope. The path traveled by the cells was analyzed using Fiji2 software.

Intestinal villi length was measured from the base to the apex using the Image J software segmented line tool. For each villus the length was subdivided into 3 equal sections (base, stem, apex), cells were counted and reported as per these subdivisions. For each subdivision, cell counts are reported as an average, by dividing the number of cells counted per subdivision by the total number of villi (*Ipmk*^*F/F*^ 40, *Ipmk*^*ΔIEC*^ 42).

#### CPT-11 treatment and tissue repair study

Mice were given a single administration of CPT-11 (Sigma) by intraperitoneal injection at 250 mg/kg in saline, in a total injection volume of 200 μl. Mice were sacrificed 4 days post-injection as described above, with both small and large intestine taken for analysis.

#### Enteroid isolation from IPMK wild type and conditional knockout mice

Intestinal enteroids were isolated using a process adapted from STEMCELL Technologies protocol “Intestinal Epithelial Enteroid Culture with IntestiCult™ Enteroid Growth Medium (Mouse).” Mice were euthanized via CO_2_ followed by surgical excision of the small intestine. Intestinal sections were gently flushed with cold DPBS and external membrane, blood vessels, and fat were removed. Intestinal segments were opened lengthwise and segmented into 2 mm sections which were then added to a 50 mL conical tube with cold DPBS. At this stage, the sections for each experimental group were combined. Intestinal fragments then underwent 15-25 cycles of washes in DPBS until the supernatant appeared clear. The DPBS was then replaced with room-temperature Gentle Cell Dissociation Reagent (GCDR) and left on a 25-rpm rocking platform for 20 minutes. Fragments were then resuspended in cold PBS with 0.1% BSA, after which the supernatant was collected and passed through a 70 μm filter. This process of resuspension and filtration was repeated to create 5 fractions; each fraction was then centrifuged with pellets resuspended in cold DMEM/F12. Samples of each fraction were evaluated by light microscopy, and those with the highest proportion of intestinal crypts were selected for plating. Selected fractions were then centrifuged and resuspended in supplemented IntestiCult™ Enteroid Growth Medium with 100 mg/mL streptomycin. The resuspension was mixed with equal parts Matrigel® and plated on a pre-warmed 24-well plate. After 10 minutes, additional growth medium was added and Enteroid cultures were left to proliferate for 7 days. At this time, the Enteroids were passaged by first homogenizing the Matrigel domes in GCDR, followed by pelleting the Enteroids. GCDR was then aspirated and the pellet was resuspended in cold DMEM/F-12, followed by a second round of centrifugation. The pellet was then homogenized in IntestiCult Enteroid Growth Medium with 100 mg/mL streptomycin, combined with Matrigel, and re-plated as previously described. For inhibitor study enteroids were isolated and plated as described above. At the time of passaging, IntestiCult Enteroid Growth Medium was swapped with media containing 1 μM GSK2334470 [PDK1 inhibitor] or AKT inhibitor VIII. Enteroids were harvested and sectioned 7 days after passaging.

#### Confocal microscopy

Images of adherent cells were acquired with a Zeiss LSM700 single-point, laser scanning confocal microscope. Histologic sections were imaged with both the Zeiss LSM700 as well as an ImageXpress Micro XLS widefield high-content analysis system. Images were analyzed with Zenlite, Imaris, and ImageJ software.

### Quantification and statistical analysis

All plots and statistical analysis were performed with Prism 8 (GraphPad) software. Statistical significance was determined by either Student's t-test (two-tailed) for 2 groups or 1-way ANOVA for multiple groups with similar sample sizes. Error bars represent standard error of the mean, and n indicates number of experimental replicates or the number of animals employed. Differences between groups were considered significant when ∗p<0.05, ∗∗p<0.01, and ∗∗∗p<0.001.

## Data Availability

• This paper does not report the original code. • This paper does not have any genomic or sequencing data. • Any additional information required to reanalyze the data reported in this paper is available from the lead contact upon request.
