# Supplementary material for: Loss of PI3k activity of inositol polyphosphate multikinase impairs PDK1-mediated AKT activation, cell migration, and intestinal homeostasis
Source: iScience. 2023 Apr 11;26(5):106623. doi: 10.1016/j.isci.2023.106623 (PMC10197106; doi:10.1016/j.isci.2023.106623)

iScience, Volume *26*

Supplemental information

Loss of PI3k activity of inositol polyphosphate multikinase impairs PDK1-mediated AKT activation, cell migration, and intestinal homeostasis

Luke Reilly, Evan R. Semenza, George Koshkaryan, Subrata Mishra, Sujan Chatterjee, Efrat Abramson, Pamela Mishra, Yoshitasu Sei, Stephen A. Wank, Mark Donowitz, Solomon H. Snyder, and Prasun Guha

SUPPLEMENTAL INFORMATION

Supplementary Figure 1. Importance of IPMK deletion on PIP production and AKT activation. Related to the Figure 1.

Supplementary Figure 2. PI3k activity of IPMK is required for activation of AKT. Related to the Figure 2.

Supplementary Figure 3. IPMK regulates cell PDK1. Related to the Figure 3.

Supplementary Figure 4. IPMK is essential for intestinal integrity. Related to the Figure 4.

Supplementary Figure 1

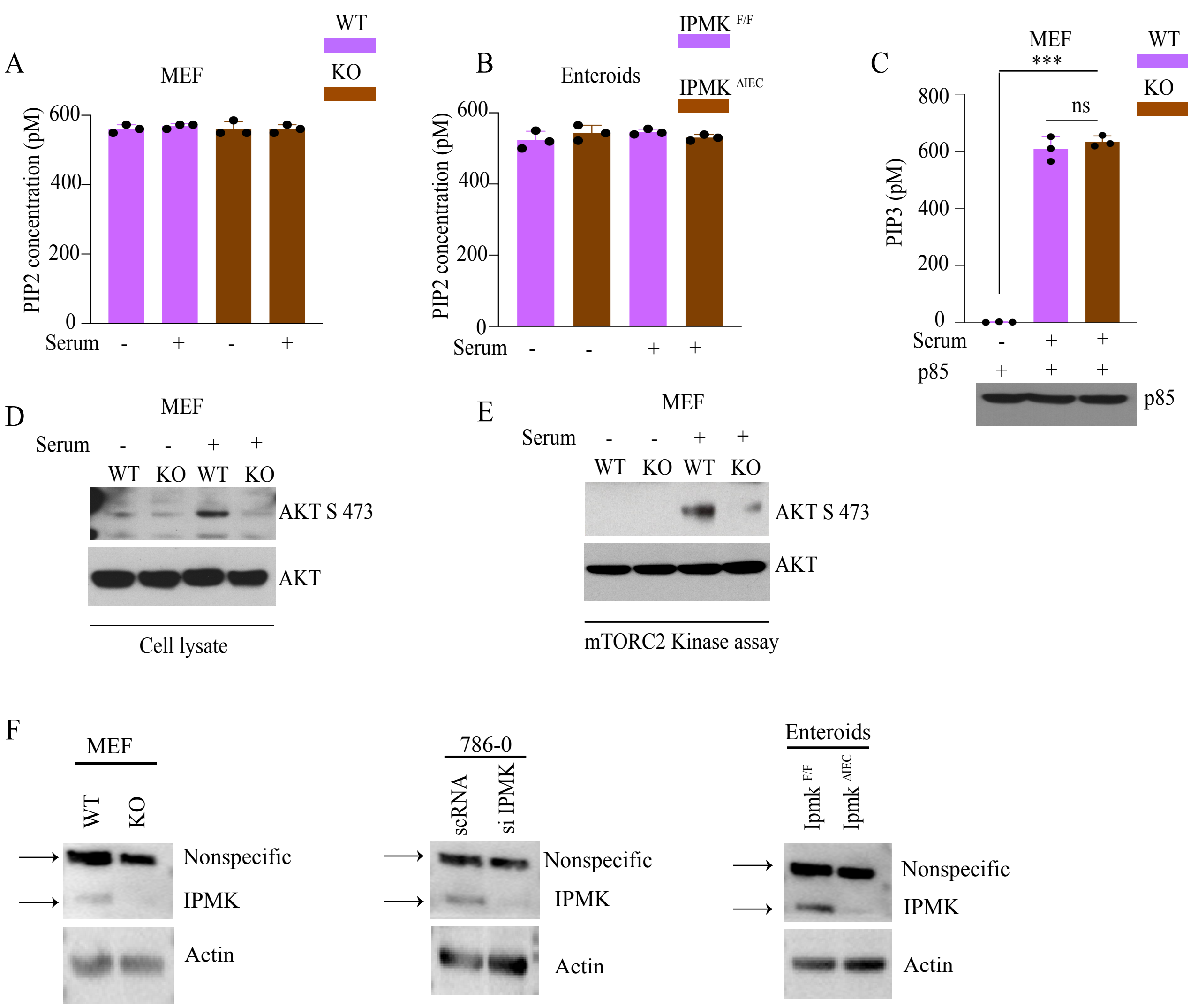

Supplementary Figure 2

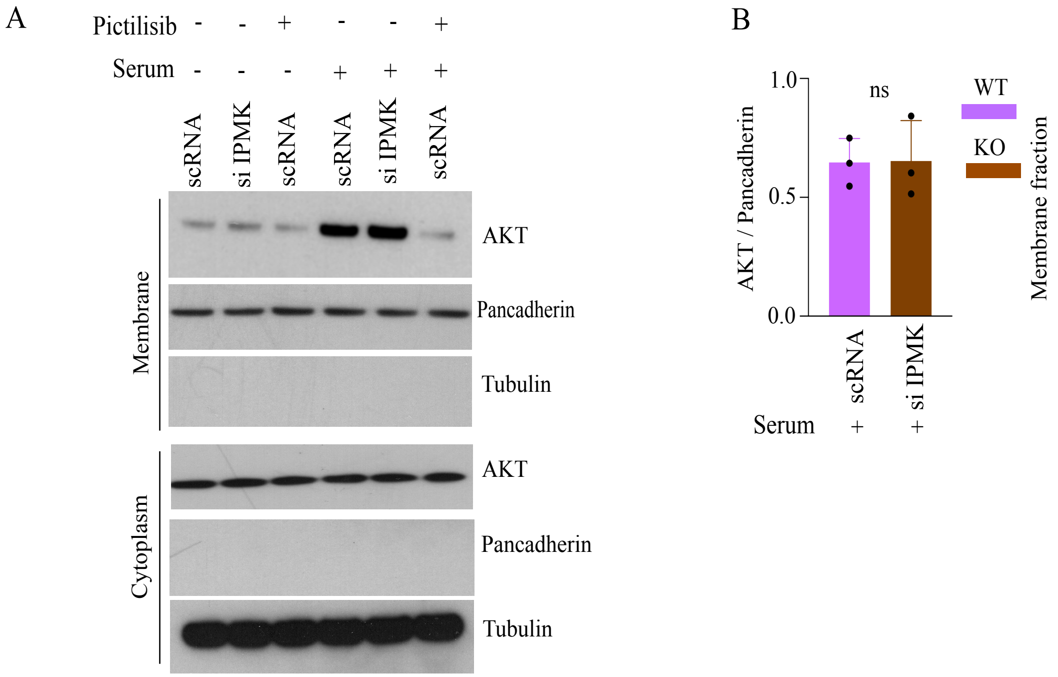

Supplementary Figure 3

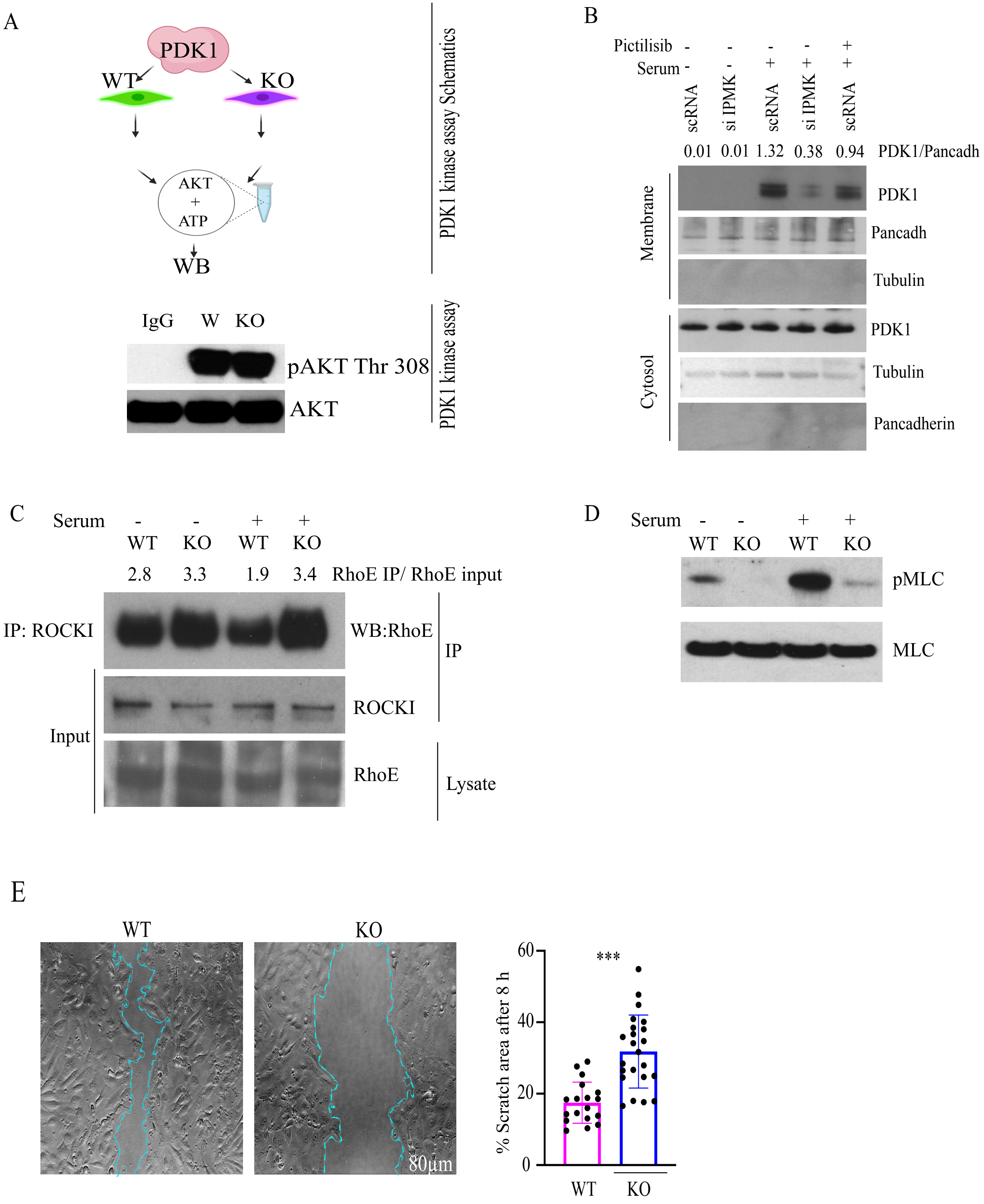

Supplementary Figure4

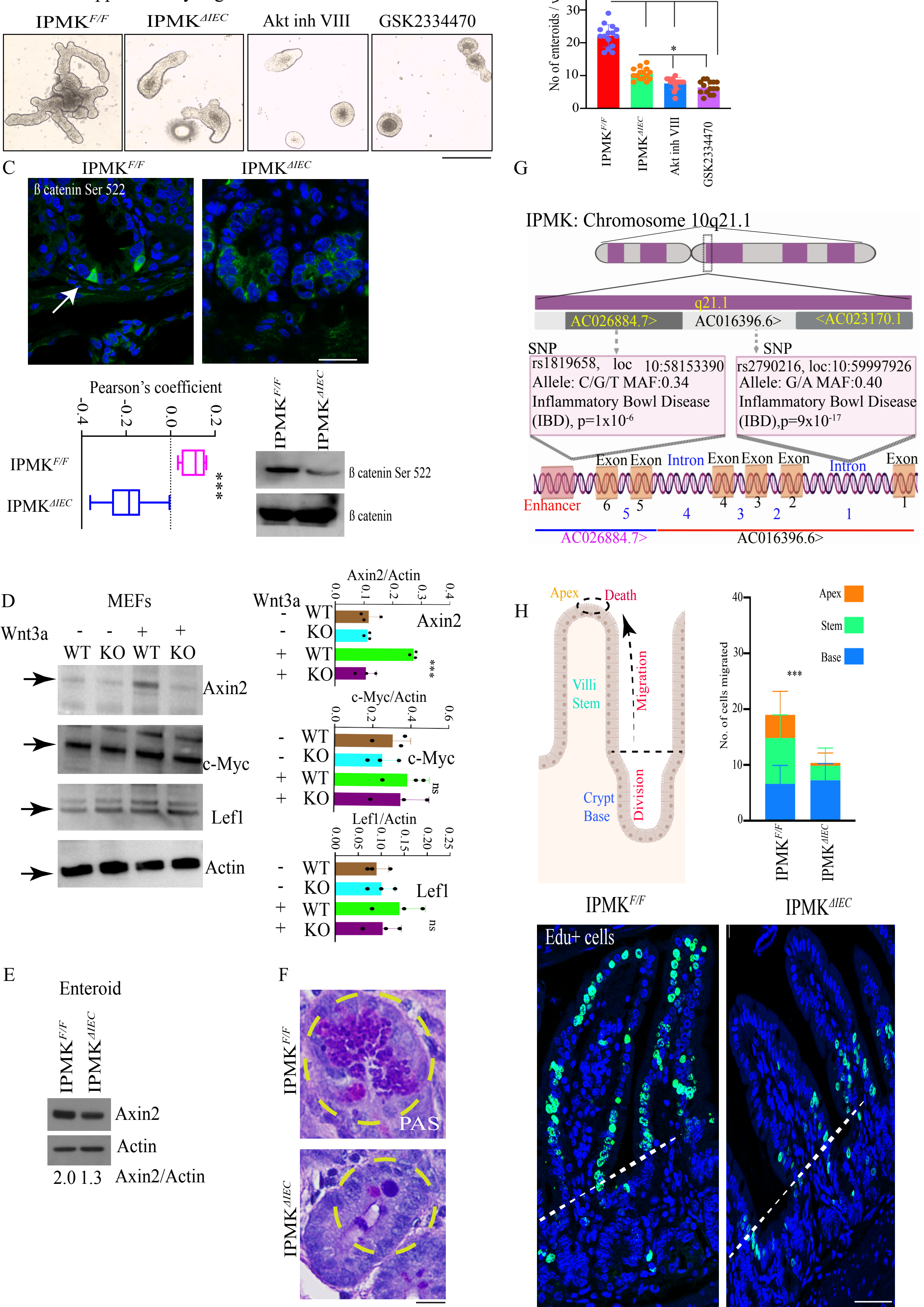

Supplement: Document S1. Figures S1–S4 [file mmc1.pdf]
